# Surgical management of complicated Stanford type A aortic dissection with subdural haematoma due to constrictive pericarditis—a case report

**DOI:** 10.3389/fcvm.2025.1583332

**Published:** 2025-08-13

**Authors:** Weibo He, Dongmei Sun, Shiyuan Yao, Yuan Wu, Linglin Fan

**Affiliations:** ^1^ Department of Cardiac Surgery, Yue Bei People’s Hospital of Shantou University, Shantou University Medical College, Shaoguan, China; ^2^ Department of Oncology, Yue Bei People’s Hospital of Shantou University, Shantou University Medical College, Shaoguan, China

**Keywords:** constrictive pericarditis, aortic dissection, surgical management, subdural haematoma, prognosis

## Abstract

Constrictive pericarditis, often resulting from pericardial adhesions secondary to chest radiotherapy or cardiac surgery, generally carries a favorable prognosis when surgical intervention is performed early. In contrast, extensive acute aortic dissection is associated with a significantly higher mortality rate, highlighting the critical importance of timely surgical intervention to improve patient outcomes. Here, we present a case involving a patient with a ruptured Stanford type A aortic dissection complicated by constrictive pericarditis, which led to the formation of a large pseudoaneurysm causing severe compression of the right atrium and markedly increasing the risk of mortality. The patient underwent successful emergency one-stage surgery and was discharged with a favorable recovery. This report uniquely highlights the management of two life-threatening conditions—type A aortic dissection and constrictive pericarditis—in a single surgical procedure, aiming to enhance patient survival and minimize complications. This case not only illustrates a successful life-saving intervention but also provides a strategic framework for cardiac surgeons, encouraging them to address complex, high-risk cases with an integrated approach.

## 1 Background

Constrictive pericarditis (CP) is a chronic inflammatory condition typically characterized by scarring, fibrosis, and calcification of the pericardium, leading to diastolic dysfunction, low cardiac output, and heart failure (1, 2). In Western countries, idiopathic pericarditis is the leading cause of CP, followed by surgery and radiotherapy, while tuberculous pericarditis remains the primary cause in developing countries and immunocompromised patients. Complete pericardiectomy is the definitive treatment for CP, with a generally favorable prognosis when performed early (3, 4). However, patients with advanced disease or ventricular systolic insufficiency face a high operative risk, with perioperative mortality rates estimated between 40% and 60% (5). Acute constrictive pericarditis normally requires rapid pericardiocentesis or pericardial debridement, but when paired with aortic dissection, the dissection can rupture at any time. Acute type A aortic dissection is one of the most aggressive and complex conditions in cardiovascular surgery (6). While surgical treatment for CP is well-established, the timing and prognosis of acute aortic dissection complicated by CP continue to pose significant challenges in clinical practice (3, 7).

## 2 Case presentation

**Figure 1:**
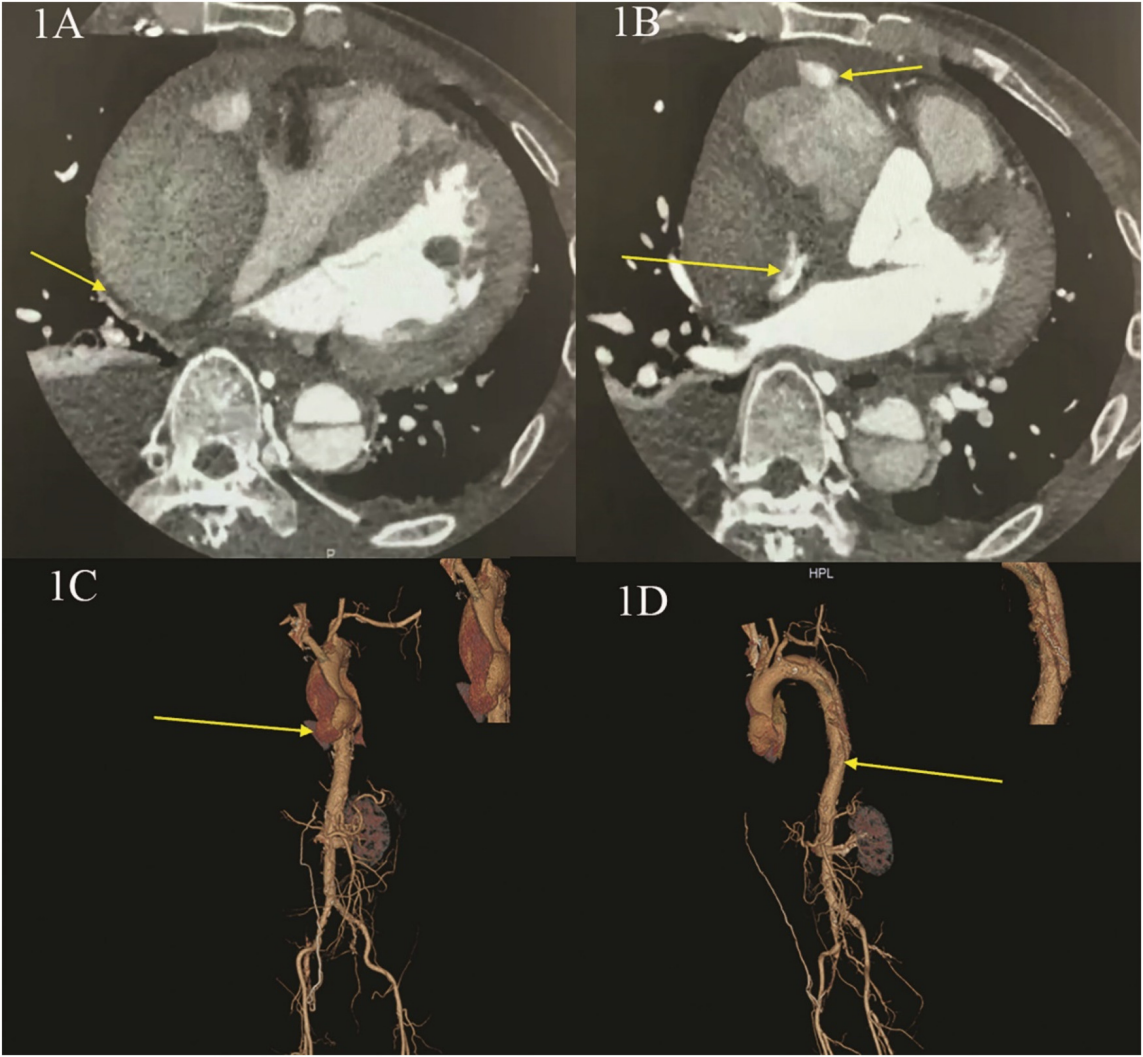
Preoperative CT angiography (CTA) of the thoracic aorta and abdominal and coronary arteries. **(A,B)** CTA shows: entrapment with aneurysm rupture in the root of ascending aorta, forming a huge pseudoaneurysm and thrombus, with obvious compression of the right heart; **(C,D)** CTA three dimensional reconstruction shows: the first rupture of aortic entrapment is located in the root of the ascending aorta, and the second rupture is located in the descending aorta.

Here, we present the case of a 52-year-old male patient who was admitted to our hospital with a 10-day history of intermittent chest and abdominal pain, which had worsened significantly over the past 24 h. Initially, the pain was tolerable and episodic; however, following a meal one day prior to admission, the pain became unbearable, characterized by a tearing and cutting sensation. Physical examination revealed no significant abnormalities. Neurological assessment, including evaluation of limb muscle strength, muscle tone, and physiological reflexes, demonstrated normal findings: limb muscle strength and tone were within normal limits, bilateral knee reflexes were present, and no pathological signs were observed. The patient's medical history included hypertension and pericardial effusion of unknown origin. Preoperative laboratory tests indicated moderate anemia, while all other parameters were within normal ranges. Computed tomography angiography (CTA) of the thoracic aorta, abdominal arteries, and coronary arteries revealed a DeBakey type I aortic dissection involving the aortic valve. The imaging findings demonstrated two rupture sites: the first located at the root of the ascending aorta and the second in the descending aorta. Additionally, there was evidence of ascending aortic root entrapment with aneurysm formation, rupture of the ascending aortic pseudoaneurysm cavity, and the development of a massive pseudoaneurysm accompanied by peripheral thrombus formation. Notably, the pseudoaneurysm caused significant compression of the adjacent right atrium and ventricle. Coronary artery evaluation indicated moderate luminal narrowing in the left anterior descending artery and mild luminal narrowing in the right coronary artery (Figures 1A,D, Supplementary Materials S1, S3, S4). Preoperative echocardiogram revealed atrial compression secondary to pericardial constriction, along with evidence of hypertensive heart disease (left ventricular hypertrophy, diastolic dysfunction). No other structural or functional abnormalities were detected (Supplementary Material S5). Based on these findings, the patient was diagnosed with type A aortic dissection complicated by chronic constrictive pericarditis.

The patient's condition was critical, as the extensive aortic dissection posed an imminent risk of further rupture, potentially leading to cardiac arrest, hemorrhagic shock, or other life-threatening complications. Given the patient's history of severe constrictive pericarditis, the risk of mortality associated with conventional surgery was exceptionally high, and the overall prognosis was poor. In this case, after thorough preoperative preparation and obtaining informed consent from the family, emergency surgical intervention was initiated. General anesthesia was induced by the anesthesiologist, followed by tracheal intubation. A median sternotomy was performed, and heparinization was administered. The presence of extensive pericardial adhesions compressing both the right atrium and the venae cavae (superior and inferior) posed significant technical challenges for establishing conventional bicaval extracorporeal circulation. This anatomical constraint substantially complicated the successful initiation of effective extracorporeal circulation. Arterial perfusion was established through the right femoral artery and the right innominate artery, while venous drainage was achieved via femoral vein and superior vena cava cannulation to initiate extracorporeal circulation. A left heart vent was placed through the right upper pulmonary vein. Upon opening the pericardium, extensive adhesions and significant thickening (up to 1 cm at its thickest point) were observed, consistent with constrictive pericarditis. A rupture site was identified at the posterior walls of the ascending aorta and aortic arch, with the aortic root rupturing into the pericardial cavity. The procedure was exceptionally challenging due to the pressurized blood from the ruptured aorta compressing the right atrium and ventricle. Additionally, adhesions were meticulously dissected to fully expose the aortic root and superior vena cava due to the complexity of the anatomy.

**Figure 2:**
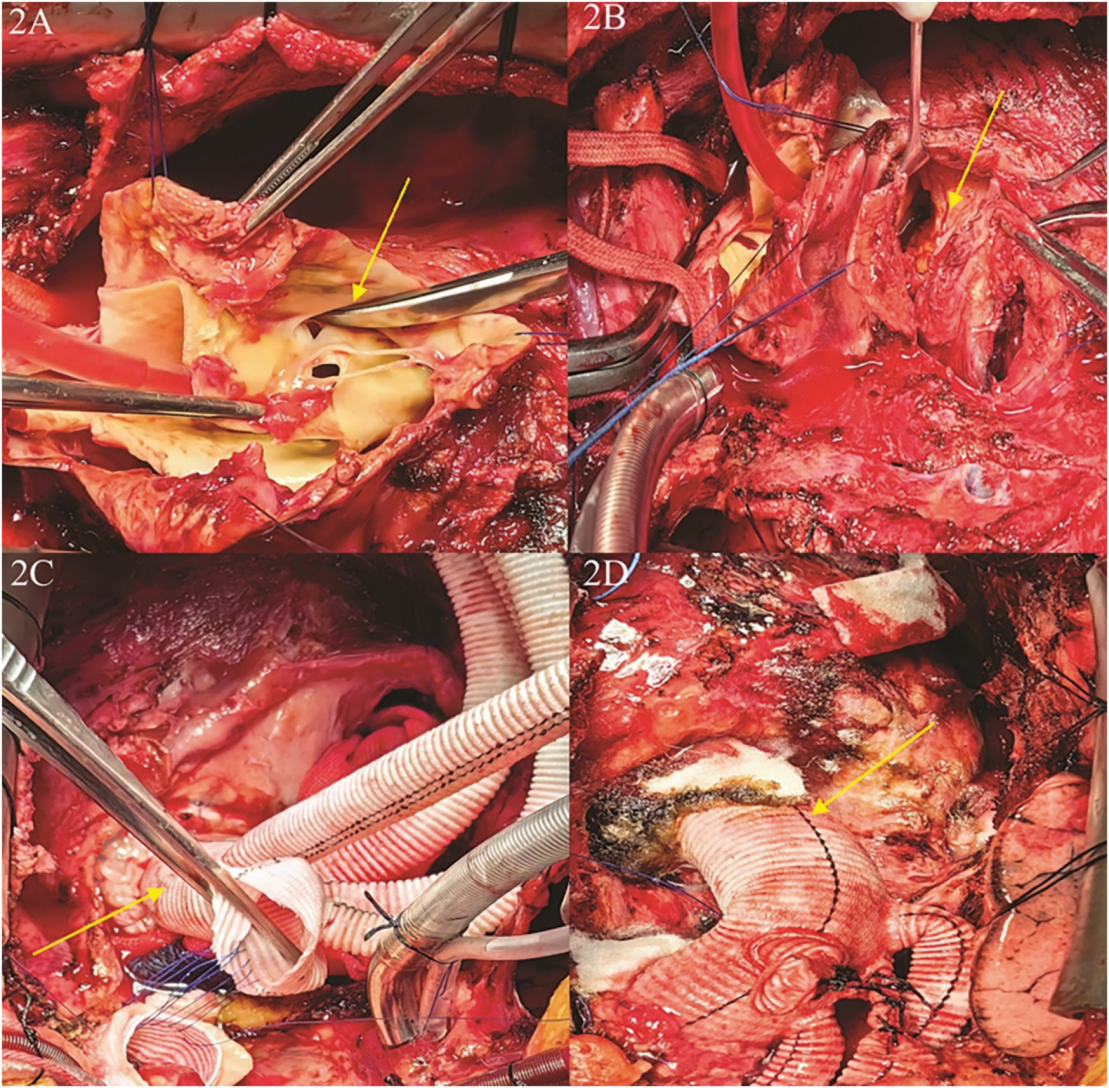
Close-up medical images of a surgical procedure involving the heart. **(A)** The first rupture of the aortic dissection was located in the root of the ascending aorta; **(B)** the first rupture of the aortic dissection ruptured into the pericardium, the patient had a combination of constrictive pericarditis, and the hematoma caused by the rupture was confined to the right atrium and right ventricle; **(C)** separation of pericardial adhesions, exposure and isolation of the aortic root, repair and reconstruction of the aortic root,replacement of the ascending aorta and the total aortic arch, and anastomosis of the artificial vessel after distal implantation of a branching stent; **(D)** completion of ascending aortic replacement + total aortic arch replacement + stenting elephant trunk surgery, and completion of narrowing pericardiectomy.

Intraoperative assessment revealed no significant involvement of the left coronary artery ostium, while the right coronary artery exhibited palpable plaque and significant stenosis, consistent with preoperative CTA findings. Aortic valve testing showed mild regurgitation, and no hematoma was observed in the brachiocephalic trunk, left common carotid artery, or left subclavian artery. A partial resection of the ascending aorta was performed, followed by artificial vascular replacement, total aortic arch replacement, and stenting of the elephant trunk. Additionally, reconstruction of the left subclavian artery, left common carotid artery, and innominate artery was carried out, along with endoluminal isolation of the descending aorta. Partial pericardial relaxation and resection were also performed, and a temporary pacemaker was implanted. The aorta was transected above the sinotubular junction, and a quadruple bifurcated artificial vessel (Intergard 24100808) was used to anastomose the proximal aorta using the sandwich technique with a combination of continuous and interrupted sutures. This approach successfully preserved the patient's aortic valve, obviating the need for Bentall procedure. During the procedure, systemic cooling was initiated until the body temperature reached 25°C. At this point, circulation was arrested, and selective cerebral perfusion was established via the head and brachial arteries, as well as bilateral common carotid arteries, at a perfusion flow rate of 5 ml/kg/min. A Cronus single-branch intraoperative stenting system (26 mm × 120 mm) was then implanted into the aortic arch and descending aorta. The stent system adhered well to the descending aortic wall, with the proximal section aligned flush with the left common carotid artery. The branching vessel was positioned into the left subclavian artery, and the quadruple bifurcated artificial vessel was continuously sutured to the stent system using 4-0 Prolene suture. Following stent placement, the aortic block clamp was opened, and rewarming was initiated. The aortic cross-clamp time was 180 min, and the total circulatory arrest time was approximately 300 min. Upon rewarming, the heart spontaneously resumed normal rhythm. Pericardiectomy was then performed to relieve obstructions in the left and right ventricular outflow tracts, as well as the superior and inferior vena cava (Figures 2A,D).

Following surgery, the patient was transferred to the intensive care unit (ICU) for further management. To prevent infection, cefuroxime sodium (1.5 g) was administered, along with cardiotonic diuretics and other symptomatic treatments. The patient regained consciousness 3 h postoperatively but subsequently developed convulsions and repeated 3 times. Physical examination revealed bilateral pupils of equal size and spherical shape, approximately 2.0 mm in diameter, with sluggish light reflex. A head CT scan identified a left frontotemporal parieto-occipital subdural hematoma and a small right parietal subdural hematoma (Supplementary Material S8). We adopted the management strategies included enhanced brain protection, mannitol for dehydration, and sodium valproate to control convulsions, and lumbar puncture to reduce intracranial pressure. The patient was extubated on postoperative day 3(During the first two postoperative days, the patient experienced one episode of convulsion on the first day and three episodes on the second day).

**Figure 3:**
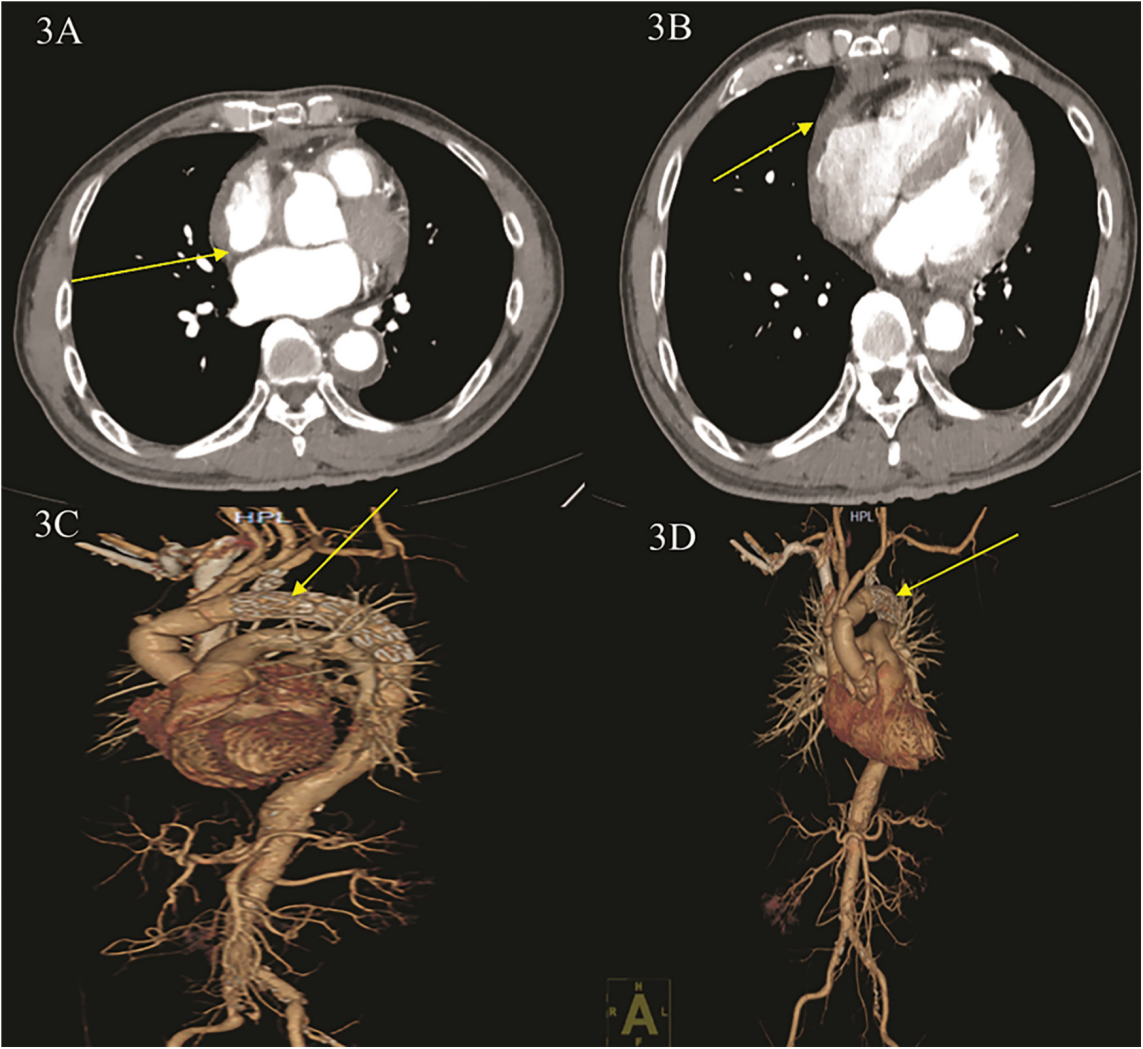
Postoperative CT angiography (CTA) of the thoracic aorta and abdominal and coronary arteries. **(A,B)** CTA shows: blood accumulation around the ascending aorta was significantly reduced, and right heart compression was reduced. **(C,D)** CTA three-dimensional reconstruction shows: ascending aortic replacement + total aortic arch replacement + stenting elephant nose after stenting, stenting position is good, no contrast leakage is seen.

The patient was transferred out of the ICU on postoperative day 5 and discharged from the hospital on postoperative day 10 (Supplementary Material S2). At the 1-month follow-up, the patient demonstrated excellent postoperative cardiac function, with an ejection fraction of 60% at 2 weeks and 65% at 1 month. Postoperative CT imaging of the thoracic and abdominal aorta confirmed normal positioning of the artificial vessels and stents (Figures 3A,D, Supplementary Materials S6, S7).

## 3 Discussion

The development of acute aortic dissection (AAD) usually results in pericardial tamponade. Pericardial tamponade is one of the most important risk factors for poor prognosis in patients with acute Aortic dissection (8, 9). The in-hospital mortality rate for AAD with pericardial tamponade is 54%, which is more than twice the mortality rate without pericardial tamponade. Therefore, aortic repair should be performed as soon as pericardial tamponade is present (6, 10). However, the pericardial systolic and diastolic dysfunction in patients with previous constrictive pericarditis predisposes them to severe pericardial occlusion during acute aortic dissection, resulting in cardiac arrest, a dramatic fall in cardiac output and arterial blood pressure, and ultimately haemodynamic disturbances.

Previous studies have used a hybrid surgical and percutaneous approach for patients who was suspected in a patient with acute coronary syndrome. The patient underwent hemiarch ascending aortic replacement with Hemashield platinum graft after resection of the ruptured aortic dissection above the sinus of valsalva the procedure was done successfully without complications (11). However, our patient had a history of unexplained pericardial effusion several years before, and the pericardium showed chronic constrictive pericarditis on intraoperative exploration, with a thickness of 1 cm at its thickest point, which, in combination with the chest pain and the onset of entrapment only a few days before, led to a combination of acute aortic dissection and chronic constrictive pericarditis. The patient had severe chronic constrictive pericarditis with palpable plaque and significant stenosis in the right coronary artery, and was unable to provide a proximal landing zone for a stent in the thoracic aorta, and a hybrid surgical and percutaneous approach was not applicable. Compared with the hybrid surgical and percutaneous two-stage surgery, the one-stage partial ascending aortic resection with artificial vascular replacement and total aortic arch artificial vascular replacement and stenting of the elephant trunk and endoluminal isolation of the descending aorta and pericardial release partial resection and implantation of a temporary pacemaker, although it is not possible to avoid the impact of extracorporeal circulation on the body, but at the same time, it also greatly reduces the plight of haemodynamic disorders in the operation. The simultaneous resolution of both thoracic and abdominal aortic tears in a single operation avoids the problems of infection and high hospitalisation costs associated with a second operation, and reduces the risk of death and heart failure.We were able to solve the patient's problem by a one-stop partial resection of the ascending aorta with prosthetic vascular replacement and total aortic arch prosthetic vascular replacement and stenting with an elephant trunk and endoluminal isolation of the descending aorta and pericardial relaxation with partial resection+ and temporary pacemaker implantation at the same time. High-risk aortic dissection repair and pericardiectomy were performed, and the patient recovered well after the operation. Compared with conventional bicaval cannulation techniques for extracorporeal circulation establishment, our findings demonstrate that establishing extracorporeal circulation through combined arterial perfusion (right femoral and innominate arteries) with venous drainage (femoral vein and superior vena cava cannulation) represents an innovative surgical approach for high-risk aortic dissection complicated by constrictive pericarditis. Furthermore, our perioperative protocol—incorporating early cerebral protection measures including mannitol-induced dehydration, valproate sodium for seizure control, and lumbar puncture to manage elevated intracranial pressure—offers a comprehensive management strategy for patients developing postoperative neurological complications. Therefore, this therapeutic approach may provide a new therapeutic strategy for the treatment of patients with such complex high-risk aortic dissection combined with chronic constrictive pericarditis.

However, there were several limitations in our study. First, our report includes specific types of patients with constrictive pericarditis combined with aortic dissection and is not fully representative of the surgical management of patients with constrictive pericarditis combined with aortic dissection; Secondly, in our patients with constrictive pericarditis combined with aortic dissection with subdural haematomas, the incidence of subdural haematomas may be correlated with a longer operative time; Finally, larger retrospective studies with larger samples will be required in the future to further statistical analyses.

In conclusion, in contrast to the 54% in-hospital mortality rate associated with AAD and tamponade, our patient's favorable outcome not only highlights the potential benefits of simultaneous intervention, but also the shortening of the extracorporeal circulation time, our case not only provides a good reference value for the treatment of such patients, but also provides a novel therapeutic idea for the clinical management of such high-risk acute aortic dissection patients.

## Data Availability

The raw data supporting the conclusions of this article will be made available by the authors, without undue reservation.
