# Supplementary material for: Surgical management of complicated Stanford type A aortic dissection with subdural haematoma due to constrictive pericarditis—a case report
Source: Front Cardiovasc Med. 2025 Aug 13;12:1583332. doi: 10.3389/fcvm.2025.1583332 (PMC12380685; doi:10.3389/fcvm.2025.1583332)
Supplement: Supplementary file 1 [file Datasheet1.pdf]

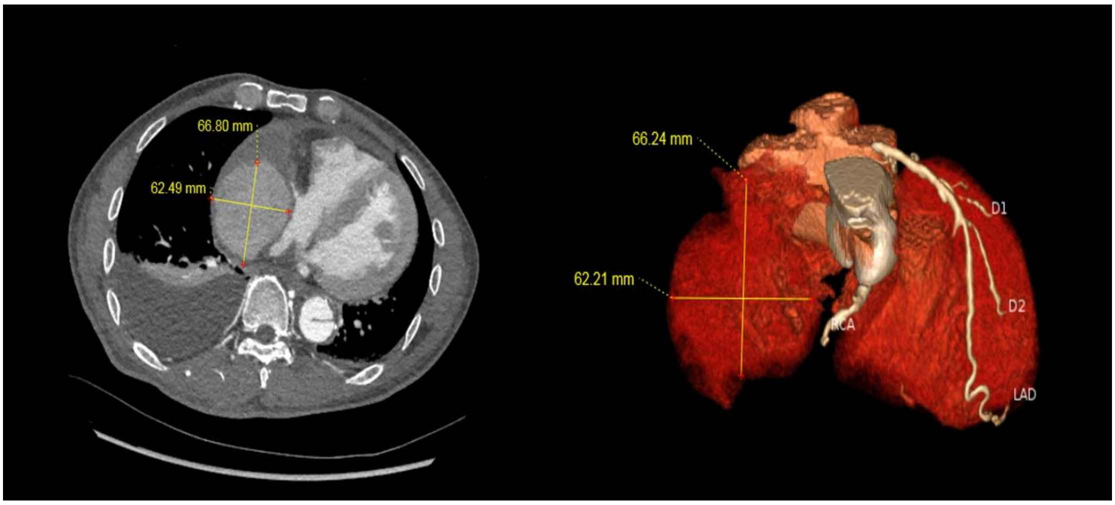

Supplemental figure 1:The size of huge pseudoaneurysm.

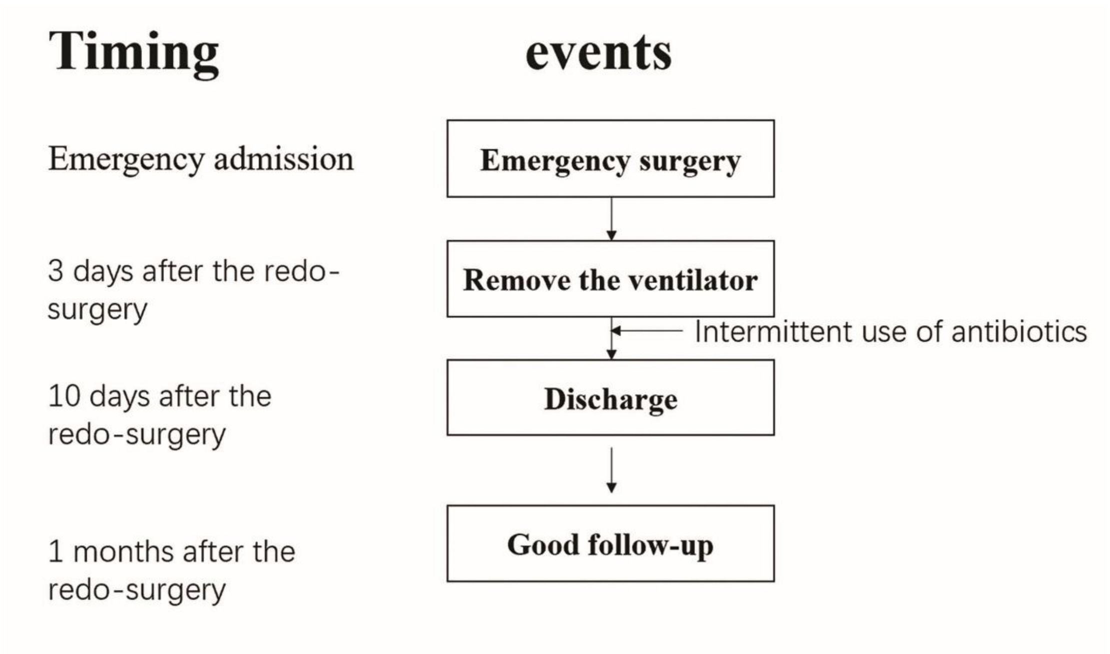

Supplemental figure 2

Flowchart of the patient's admission to the hospital for emergency surgical treatment;

Ventilator was removed 3 days postoperatively; Recovered and discharged 10 days postoperatively; The patient was in good condition at 1 month postoperative follow-up;

Supplemental video 1 and 2

Preoperative CT angiography (CTA) of the thoracic aorta and abdominal and coronary arteries .

Supplemental video 3

Preoperative echocardiogram.

Supplemental video 4 and 5

Postoperative CT angiography (CTA) of the thoracic aorta and abdominal and coronary arteries .

Supplemental video 6

Postoperative Cranial computed tomography (CT)
